# Sex-Related Differences in Lifestyle Factors Affecting Multiple Sclerosis Susceptibility and Disease Progression

**DOI:** 10.3390/brainsci15101097

**Published:** 2025-10-11

**Authors:** Elena Barbuti, Claudia Piervincenzi, Serena Ruggieri, Maria Petracca

**Affiliations:** 1 Department of Human Neurosciences, Sapienza University of Rome, 00185 Rome, Italy; elena.barbuti@uniroma1.it (E.B.); claudia.piervincenzi@uniroma1.it (C.P.); 2 Department of Neurosciences, San Camillo Forlanini Hospital, 00152 Rome, Italy; serena.ruggieri@gmail.com

**Keywords:** multiple sclerosis, review, disease progression, sex, gender, smoking, vitamin D, obesity, estrogens, EBV

## Abstract

Multiple sclerosis (MS) is a chronic autoimmune disease of the central nervous system that affects women more frequently than men. This sex gap has widened over the past century, and appears to be shaped by lifestyle factors more than biological factors. This narrative review examines the evidence for sex-specific differences in lifestyle risk factors and their impact on both MS susceptibility and disease progression, with implications for diagnosis, monitoring, and treatment. Smoking, obesity, vitamin D deficiency, ultraviolet radiation exposure, and Epstein–Barr virus infection all interact with sex-related biological pathways to influence MS risk. Women appear to be more vulnerable to the pathogenic effects of smoking and obesity, both independently and in synergy with genetic risk alleles, while vitamin D and UV exposure confer stronger protective effects in females than in males. EBV infection also exhibits sex-dependent immune responses, shaped by hormonal regulation and host–virus genetic interactions. Sex-related lifestyle factors also modulate MS progression. Women experience more inflammatory activity and relapses, whereas men more often develop a progressive phenotype with greater neurodegeneration. Hormonal changes during female reproductive phases, such as pregnancy, breastfeeding, menopause, and hormone-based therapies, critically influence disease activity and progression in MS. Obesity, smoking, vitamin D status, diet, and gut microbiota further interact with sex hormones and genetic background, contributing to variable disease trajectories, also modulated by social determinants such as education level. These findings underscore the need to integrate into clinical practice the evaluation of lifestyle factors in a sex-specific way for diagnosis, monitoring, and treatment of MS.

## 1. Introduction

Multiple sclerosis (MS) is an autoimmune disease of the CNS that is significantly more prevalent in females, with an overall female-to-male ratio of 3:1 [1]. Estimates from the Global Burden of Disease Study 2019 suggest that females exhibit a higher point prevalence and a greater total number of prevalent cases than males across all age ranges, except for the 5–9 age range [2]. Also, in the context of rising MS incidence, observed in longitudinal studies until 2000 [3], a disproportionate increase in females has been reported, with females being twice as likely to be diagnosed with MS than males [4,5]. Indeed, female-to-male ratio has risen over the last century, although recent data show a stabilization in certain regions [6].

Sex-related differences in MS susceptibility and progression have been consistently reported as a consequence of genetic, epigenetic and hormonal factors [7,8,9]. In particular, the rising MS incidence among females, which cannot be explained solely by genetic predisposition, points to sex-specific environmental or epigenetic influences [10]. Lifestyle, in terms of exposure to environmental risk factors, daily habits and life choices that could affect MS onset and modulate its clinical evolution, has been the object of a growing interest both in the scientific community and in the wider public [11,12,13]. In this light, the observed differences might be driven not only by sex-related biological factors, but also by gender-related behaviors and activities [14]. Distinguishing between the impact of sex and gender, however, is complex, and, to the best of our knowledge, no study has been conducted so far to specifically explore the independent role of gender in MS onset and course. As a result, in this work, the term “sex-differences” encompasses both biological and behavioral aspects. In this narrative review, we summarize the relevant findings on sex-related differences in lifestyle factors affecting MS susceptibility and progression, highlighting their implications for disease diagnosis, monitoring and treatment.

## 2. Sex-Related Differences in Lifestyle Factors Affecting Susceptibility

Several lifestyle factors, including smoking, obesity, vitamin D deficiency, ultraviolet radiation (UVR) exposure and Epstein–Barr Virus (EBV) infection could contribute to MS susceptibility in a sex-specific way (see Table 1 and Table 2) [15]. Insufficient and poor-quality sleep, by enhancing immune activation and impairing glymphatic clearance, may exacerbate neuroinflammation and has recently been identified as a risk factor for MS development; however, sex-specific differences in this context have not yet been investigated [16].

### 2.1. Smoking and Other Inhalant Irritants

Cigarette smoking has been consistently associated with an increased risk of developing MS in a dose-dependent manner [42,43]. This effect is attributed to harmful components in tobacco smoke, such as free radicals, cyanates, and carbon monoxide, which promote oxidative stress, lung inflammation, and alterations in both innate and adaptive immune responses, ultimately leading to increased autoimmunity [43,44,45]. Nicotine may have a protective effect against MS, as suggested by the lower risk associated with non-inhaled tobacco (snuff), possibly via activation of α7 acetylcholine receptors on immune cells, which promotes a shift toward anti-inflammatory Th2 responses [46,47]. The hypothesis that lung irritation itself, rather than nicotine, drives increased MS risk has led to consideration of other inhaled irritants such as passive smoking, air pollution and organic solvent exposure as possible triggers for MS development [45].

Studies investigating sex differences in smoking as a risk factor for MS have reported conflicting results, with either smoking males or females exhibiting a higher risk of developing MS [21,48]. Over the past decade, smoking habits have changed significantly, with smoking becoming more common among women and less common among men, leading to an increased female-to-male smoking ratio. A correlation has been observed between these changing smoking patterns and the rising female-to-male ratio in MS incidence, suggesting that smoking may be contributing to the growing sex disparity in new MS cases [22]. However, the difference in susceptibility to smoking-related MS risk appears to depend not only on gender-related behavioral changes, but also on biological sex differences. For example, serum levels of nicotine metabolites have been more strongly associated with MS risk in women than in men, indicating a greater female sensitivity to the harmful effects of smoking [23]. In a recent Iranian study, while men were more frequently smokers than women and smoked a higher number of cigarettes, women exhibited a shorter interval between smoking initiation and MS onset, suggesting a higher biological vulnerability to smoking-related damage [24]. Regarding passive smoking, current evidence does not support a sex-specific difference in MS risk [25], while no study to date has assessed a sex effect on air pollution or organic solvents risk of developing MS.

Women could be more susceptible to the pathogenic effects of smoking. In general, women have more robust innate and adaptive immune responses, with higher antibody production and stronger pro-inflammatory reactions, which account for their increased susceptibility to inflammatory and autoimmune diseases [49]. These differences arise from both genetic factors, including chromosome X- and Y-linked genes, microRNAs, long non-coding RNA and genetic polymorphisms, that regulate immune function, and hormonal influences, particularly relevant after puberty and before reproductive senescence [50]. Smoking interacts with two established genetic risk factors for MS, carriage of HLA-DRB1 15:01, more frequently observed in females [51,52], and absence of HLA-A02, which together raise MS risk by a factor of 2.8, compared to 1.4 in individuals without genetic predisposition [53]. Smoking also induces dose-dependent and reversible epigenetic changes, such as DNA methylation, which are more pronounced in female MS patients carrying the HLA-DRB1 15:01 allele and lacking HLA-A02, compared to the general MS population [54]. Finally, smoking exerts anti-estrogenic effects, likely by accelerating estradiol metabolism and reducing its bioavailability in women, while inconsistently affecting androgen levels in men [55,56].

### 2.2. Obesity

Obesity, whether in childhood, adolescence, or young adulthood, has been associated with an increased risk of developing MS or clinically isolated syndrome (CIS) [26,57]. The main proposed mechanisms include lower serum levels of 25-hydroxyvitamin D [25(OH)D] in obese individuals, due to its sequestration in excess adipose tissue and altered metabolism, chronic low-grade inflammation driven by leptin and other adipokines, promoting enhanced T helper 1 (Th1) responses and reduced regulatory Th17 cells, gut microbiota dysfunction, and, in females, earlier onset of puberty [57,58,59,60,61]. Lower vitamin D concentrations have been found in females in comparison to males across different body mass index (BMI), and were correlated with the higher amount of fat mass in females [62].

Regarding sex-related differences, in a cohort of children under 18 years of age (n = 75), obesity was significantly associated with an increased risk of pediatric MS/CIS in girls, but not in boys [26]. Similarly, a Swedish study found no association between adolescent BMI and future MS risk in males [31]. A one-unit increase in BMI z-score was associated with an increased risk of MS in girls aged 7–13, whereas the association in boys was weaker and only significant between ages 8 and 10 [27]. However, other studies have reported comparable associations between childhood obesity and MS risk in both sexes [28,29]. In adult males, the association between obesity and MS risk appears attenuated or absent [57], while it is more consistently observed in females [30].

Obesity was shown to alter pro-inflammatory proteomic signatures in a sex-dependent manner, both in MS patients and in healthy controls [63]. Women, both with and without MS, exhibited a greater number of upregulated proteins related to Th1, IL-17, and inflammatory-related pathways, suggesting a stronger obesity-driven amplification of autoimmune responses [63]. Moreover, as with smoking, adolescent obesity interacted with carriage of HLA-DRB1 15:01 and absence of HLA-A02, or with a history of late infectious mononucleosis (IM), resulting in about a 14-fold increased risk of developing MS [64,65]. Finally, obesity has been associated with early menarche, which itself is a risk factor for MS in women [35].

### 2.3. Vitamin D Deficiency and UVR Exposure

Vitamin D deficiency and low sun exposure have been identified as independent risk factors for MS, supported by the rising prevalence of the disease at higher latitudes [58]. Sunlight exposure promotes the photoconversion of 7-dehydrocholesterol to vitamin D (cholecalciferol) in the skin, making it difficult to fully disentangle the individual contributions of the two factors. Vitamin D, also obtained through diet, has been shown to reduce MS risk, particularly in fair-skinned individuals, whereas sun exposure, but not vitamin D, was associated with MS risk in Black and Hispanic individuals [66,67]. A diet rich in fatty fish, a natural source of vitamin D, has also been associated with lower MS risk in people with limited sun exposure [68]. In animal models, UVR reduced the risk of experimental autoimmune encephalomyelitis (EAE) independently of vitamin D production [69].

Vitamin D has been linked to immunomodulatory effects, including enhanced activity of CD4^+^ regulatory T cells (Tregs), increased IL-10 production, and inhibition of both the differentiation and migration of Th17 cells [70,71,72]. Similarly, UVR exposure can induce regulatory T cells and immunoregulatory molecules while reducing TNF production [73].

Regarding sex-specific effects, higher circulating levels of 25(OH)D have been associated with a lower incidence of MS only in women [32]. In animal models, estrogens interact with Vitamin D3 to confer resistance to EAE in terms of both reduced incidence, longer time to disease onset and reduced severity [17]. Vitamin D3 inhibited EAE development in intact female mice, but not in ovariectomized females or males [74]. Low-dose estradiol (E2) alone did not prevent EAE, but restored vitamin D_3_-mediated protection in ovariectomized females when combined with vitamin D [17]. In male mice, E2 did not enable vitamin D_3_ to prevent EAE.

In both MS patients and healthy individuals, vitamin D_3_ has shown stronger immunomodulatory effects in females than in males [75]. Estrogens are thought to contribute to vitamin D accumulation by decreasing CYP24A1 gene expression, which encodes the 1,25-dihydroxyvitamin D(3)-inactivating enzyme, and by increasing of vitamin D receptor expression in the CNS, resulting in a more potent anti-inflammatory effect [17,75]. CD4^+^ T cells mediate the synergistic effects of estradiol and vitamin D3 in promoting regulatory T cell development and immune tolerance; disruption of this pathway may impair Treg differentiation and increase MS risk, especially in women [76]. Moreover, the effect of vitamin D on EAE susceptibility is influenced by both sex and genetic background, as demonstrated by a study on chromosome substitution (consomic) mouse models, incorporating the genetic diversity of wild-derived PWD/PhJ mice. Protective effects of vitamin D against EAE risk were observed only in females of certain genotypes. The findings suggest that vitamin D’s impact on MS risk is determined by complex gene-by-sex interactions [18].

UVR exposure has also been shown to reduce MS risk more in females than males, consistent with an increased female-to-male ratio at higher latitudes [33,77]. In children, greater time spent outdoors was associated with reduced risk of developing pediatric-onset MS, but sex differences were not evaluated [78]. Additionally, UVR seem to be implicated in sex hormone production with a possible impact on immunological changes [79].

### 2.4. EBV Infection

EBV is a human herpesvirus that causes infection and persists in a latent form in B lymphocytes. EBV has been inconsistently reported in MS demyelinating lesions in neuropathological studies [80,81]. In young males on active duty, the risk of MS increased 32-fold after infection with EBV, but not after cytomegalovirus infection [82]. Serum levels of neurofilament light chain, a biomarker of neuroaxonal degeneration, increased only after EBV seroconversion, indicating that EBV infection preceded the first pathological signs underlying MS and suggesting EBV infection as the leading cause of MS [82]. Several studies have noted a difference in the timing of EBV infection with respect to MS risk, pointing to infection in adolescence or early adulthood as the main risk factor for MS [83,84]. This hypothesis was confirmed by a study that demonstrated that having siblings protects against IM by occasionally preventing delayed primary EBV infection, with its associated high risk of IM [85]. It is not clear why delaying primary EBV infection should increase MS risk, but a role of sex hormones, whose levels change during puberty, has been suggested, with potential influence on immune responses [83]. Another study found that the time from IM, as a temporal marker of EBV infection, to MS onset (IM-to-MS delay) decreased with increasing age [34]. Stratifying the analysis by sex and Epstein–Barr nuclear antigen 1 (EBNA1) levels, males and high-titer subpopulations showed a shorter IM-to MS delay, further reducing from childhood to adult infection, while females and low-titer populations had a longer IM-to-MS interval, especially in early childhood, significantly shortening from childhood to adulthood, especially beyond puberty [34].

Females are more frequently EBV-seropositive and present higher levels of IgG EBV- reactive antibodies, but a lower anti-EBV IgA response than males, suggesting that men may experience a greater frequency of EBV reinfection or reactivation, while females have a more robust initial response to EBV [86,87].

Estradiol enhances both T-helper 1 and T-helper 2 immune responses and promotes humoral immunity through estrogen receptor binding, while androgens suppress immune cell activity, reduce pro-inflammatory responses, and inhibit antiviral cytokines [49]. MS risk gene expression in EBV–infected B cells was shown to be modulated in a sex-specific manner, with estradiol influencing gene expression, EBV DNA copy number and EBNA2 expression and cell proliferation [19]. These findings suggest that estrogen-mediated regulation of MS risk loci may contribute to sex differences in the immune response to EBV and partially explain the higher incidence of MS in females. A reduction in the protein CD40, implicated in tolerance mechanisms and affinity maturation processes, was found in endogenous-EBV-infected cells in MS patients relative to healthy donors, but not in cells infected with the recombinant EBV strain B95.8 [20]. The reduced CD40 expression was more pronounced in MS females than in healthy females and was almost significantly lower in MS females than in MS males. The difference was not attributable to host-associated SNPs for CD40. Moreover, EBNA2 1.2 allele increased the risk of MS by 2.5-fold. These findings underline that host genetic susceptibility and EBV genetic variability could converge on shared, disease-specific mechanisms relevant to MS pathogenesis, possibly in a sex-dependent manner. Synergistic effects of environmental and genetic risk factors could further contribute to increasing susceptibility to MS. HLA-DRB1 15:01, associated with female sex, conferred a 2.9-fold stronger risk of MS in individuals with a history of IM [88] or childhood EBV infection [89]. In pediatric MS, an additive interaction was found between prior EBV exposure and the GG genotype of the risk variant rs2255214 in CD86: sex differences were not evaluated [89]. Conversely, a negative interaction between smoking and EBV infection or between EBV and organic solvents could underlie competing pathogenic pathways: unfortunately, the role of sex in environmental factors interplay for MS risk has not yet been evaluated [90].

### 2.5. Hormonal, Gender-Related and Occupational Factors

Puberty appears to be a critical window in disease susceptibility, as earlier menarche has been associated with increased MS risk and earlier onset [35,36,91,92,93]. Hormonal changes such as a rise in estrogen levels, could account for the female-specific increase in MS risk after puberty [94]. In pediatric-onset MS, MS occurs equally in both genders in pre-puberal age, while the female/male ratio increases in post-pubertal age [95]. Exposure to the previously cited environmental risk factors associated with MS during the post-pubertal years, e.g., in presence of an increase in estrogen levels, could synergistically increase the risk, triggering the development of MS. However, the HLA-DRB1*1501 risk allele for MS was found to inversely correlate with age at menarche, highlighting the complexity in the association between hormonal regulation and genetic risk in determining age at MS onset [96].

The evaluation of hormonal contraceptive use as a risk factor for MS development has yielded conflicting results in large retrospective cohorts, showing either reduced risk, no association and increased risk, especially in nulliparous women, in relation to higher duration and earlier age at initiation of contraception [37,38,39,40,41]. Interestingly, a stronger hazard association between hormonal contraceptive use and MS risk was found in women with a low MS polygenic risk score [38]. Further research is required before any conclusion can be drawn.

Changes in women’s status in recent decades could have contributed to an increased susceptibility to environmental risk factors. As a result of urbanization and inclusion in education and work activities, women, like men, spend more time indoors and make different reproductive choices (fewer pregnancies, greater use of oral contraception) in comparison to the past [97,98].

Occupation has also been shown to affect MS risk, with agricultural and offshore workers, hairdressers and workers exposed to toxic fumes or pesticides and low-frequency magnetic fields being associated with a higher MS risk [99]. A higher prevalence of men was acknowledged in occupations considered at increased risk. In pediatric MS, having a father in a gardening-related occupation or any use of plant-related pesticides in the household from three months before pregnancy through the first year of life was associated with an increased risk of pediatric MS, after adjusting for age, sex, race, and ethnicity [100].

## 3. Sex-Related Differences in Lifestyle Factors Affecting Disease Progression

Women and men with MS present marked differences in the disease course, in terms of disease activity and progression, and exhibit distinct MRI features. Women tend to experience more frequent relapses and show a higher number of gadolinium-enhancing lesions on MRI [8,92,97,101,102,103,104,105,106,107,108,109,110,111], while men are more likely to present with a progressive phenotype and accumulate disability more rapidly, leading to a more severe disease course [7,97,104,112]. Men also show greater deep gray matter atrophy and an increased risk of cognitive decline [113]. These differences are influenced by sex-specific genetic and lifestyle factors. Separating sex chromosome effects from sex hormones is challenging, but possible in the EAE four core genotypes model. XY CNS mice showed more severe disease and neuropathology than XX, implicating sex chromosome–linked genes in neurodegeneration [114]. Interestingly, however, human studies analyzing longitudinal changes in MRI markers of inflammation and neurodegeneration have mostly failed to confirm between-sex differences reported in cross-sectional studies, with the exception of intralesional damage in males, highlighting the dynamic nature of such differences, likely depending upon life stages and individual choices [115,116,117,118,119]. Sex differences in lifestyle factors affecting disease progression are summarized in Table 3 and Table 4.

### 3.1. Hormonal Exposure

In MS, sex hormones strongly influence both immune regulation and neuroprotection [79]. Estrogens have dose-dependent effects: in EAE low levels of estradiol may promote inflammation, whereas higher levels of estriol, predominant in pregnancy, shift immunity toward a Th2 profile and increase Tregs [97]. Progesterone similarly exerts anti-inflammatory activity by reducing dendritic cell activation and promoting Th2 responses [97]. Testosterone has also been shown to reduce inflammation, with supplementation in castrated mice improving clinical outcomes [97,184]. Beyond immunomodulation, estrogens, progesterone, and testosterone all contribute to neuroprotection and remyelination in the CNS [120,122,123,124,193]. To reconcile the higher MS prevalence but more favorable outcomes in females, it has been proposed that female sex promotes disease via systemic immunological effects, whereas estrogens exert protective actions on the CNS [194].

The study of reproductive transition in women allows for a better understanding of the role of hormonal fluctuations in characterizing the MS disease course.

**Pregnancy** significantly affects MS disease activity. Relapse rates drop markedly during pregnancy—especially in the third trimester—with reductions of up to 70% [8,128,129,130,195], followed by a rebound in the postpartum period [128,129,130,196], during which MRI often shows increased inflammatory activity [91,131,132,133]. Some studies, but not all, have also suggested a protective effect of pregnancy on long-term disability progression [92,134,135,136,137,138,139,197]. While it has been hypothesized that women with more severe disease may choose not to have children due to caregiving concerns, this alone does not appear to account for the observed effects [97]. Hormonal changes during pregnancy, particularly the rise in estriol and progesterone levels, and the subsequent drop in hormonal levels in post-partum, are likely the main contributors to these specific changes in disability accrual. Based on this, the therapeutic potential of estriol or progestins has been explored in non-pregnant women, but results so far have been mixed [173,174] (see Treatment). Immunological changes during pregnancy in MS include a shift from a pro-inflammatory (Th1, Th17) to an anti-inflammatory (Th2/Treg) profile, with a down-regulation of inflammatory chemokines, upregulation of immunomodulatory molecules and expansion of regulatory T cells to achieve immune tolerance towards the fetus. Hormonal replacement could not be able to mimic the complex immunological mechanisms underlying the protective effect of pregnancy.

**Breastfeeding**, especially exclusive breastfeeding, has been associated with a reduced risk of postpartum relapse in women with MS [9,140,141,142,143,144,145], although not all studies have confirmed this association [128,146,147,148,149,150,151,152]. Hormonal changes associated with breastfeeding are high prolactin levels and suppression of pulsatile gonadotropin-releasing hormone and luteinizing hormone, with resulting lactational amenorrhea. The latter could be associated with a decline of TNF-alfa, a pro-inflammatory cytokine that increases during menses [198]. Indeed, an earlier return of menses, which could be accelerated in non-exclusive breastfeeding compared to exclusive breastfeeding, was associated with a higher risk of relapse in the first 6 months post-partum [140].

We acknowledge that the association between breastfeeding and postpartum relapse risk could be biased by women with more severe disease being more prone to forego breastfeeding in order to re-assume MS treatment. However, real-world data support this protective effect, especially in women with low to moderate disease activity, reinforcing the importance of integrating breastfeeding into individualized postpartum management whenever clinically feasible [153].

**Menopause** has been associated with mild disease worsening and more significant gray matter loss in most studies, likely related to the decrease in estrogen levels [154,155,156,157,158]. Menopausal changes may lead women to a disease course more similar to that of men, with a lower relapse rate, greater disability progression and neurodegeneration [97,159,160,161]. While the impact of **hormonal replacement therapy (HRT)** on controlling menopausal symptoms and preventing fractures is well-established, its benefit on specific MS clinical outcomes (e.g., Expanded Disability Status Scale (EDSS) worsening, new T2 lesions or gadolinium-enhancing lesions at MRI) is more controversial [162,163,164,165,199]. Symptoms of MS and menopause can frequently overlap, including disturbances in cognition, mood, sleep, and bladder function, making it challenging to distinguish menopausal-driven from age-associated or MS-specific disturbances [200]. Moreover, HRT increases the risk of breast cancer, thromboembolic events, and stroke [201]. In EAE, estradiol was shown to enhance astrocyte numbers and promote remyelination by oligodendrocytes, with a potential role in preventing neurodegeneration [120]. However, HRT protocols for women were not specifically designed for neuroprotection [194]. In healthy women, menopause induces cognitive domain-specific deficits and accelerates regional atrophy on MRI: an improvement in HRT schedule and clinical/MRI outcomes evaluated could unmask the HRT effect in MS [194].

**Assisted reproductive technologies** (ART), particularly gonadotropin-releasing hormone (GnRH) agonists, have been associated with increased relapse risk and MRI activity in the months following treatment [166,167,168,169]. Proposed mechanisms include stress associated with the procedure, as well as the effects of GnRH agonists on the B and T cell proliferation, gene transcription, and migration; furthermore, protocols using GnRH agonists are longer, more aggressive and use higher hormonal doses than protocols using antagonists [169]. However, recent data from larger multicentric cohorts found no increased risk of relapse after in vitro fertilization (IVF) neither with GnRH agonists nor with GnRH antagonists, with maintenance of disease-modifying therapies until IVF as a determining factor in reducing the risk of relapse [170,171,172]. It is advisable to plan infertility treatments during disease stability, with close coordination between neurologists and fertility specialists to optimize MS care and ART outcomes.

Although studies have reported conflicting effects on MS risk, **oral contraceptives (OC)** do not appear to negatively affect the long-term prognosis of the disease [10,174,175,176,177]. Indeed, use of OC prior to MS diagnosis has been associated with a 26% lower risk of progression independent of relapse activity (PIRA), and a delay of approximately 2.5 years in time to first PIRA event [178]. Oral contraceptive users presented lower disability (EDSS, MS severity score—MSSS) and lower risk of converting to secondary progressive (SPMS) than non-OC users [176,179]. In a randomized clinical trial, IFN-β plus ethinylstradiol and desogestrel decreased the cumulative number of active brain MRI lesions compared with IFN-β alone in RRMS women [180]. A trend towards less MRI inflammatory activity has been shown for continuous OC relative cyclic OC [181]. Interestingly, OC were associated with milder disability (EDSS, MSSS) and decreased adiponectin blood levels in RRMS women independently of BMI: OC use seemed to mitigate the correlation between adiponectin levels and disease severity, suggesting a complex interaction between sex steroids, adipose metabolism and MS progression [182].

In men, hormonal status also appears to influence disease course: low serum **testosterone** levels have been reported in male patients with MS and have been associated with greater physical and cognitive disability [7,91,97,183]. Testosterone therapy has been shown to slow brain atrophy rate and improve cognitive outcomes [185]. In male mice, myelin repair was dependent on the presence of testes and testosterone, leading to an increase in astrocytes expressing CXCR4 and recruitment of myelin-forming oligodendrocytes [122].

In addition to these sex-related hormonal dynamics, recent evidence suggests that **gender-affirming hormone therapy (GAHT)** may also influence MS disease course. In transgender individuals with MS, both feminizing and masculinizing GAHT have been linked to increased clinical and radiological disease activity, with feminizing GAHT in particular associated with earlier progression and greater disability [187]. GAHT creates a distinct chromosome–endocrine profile that highlights the relative roles of chromosomal and hormonal sex in immunity changes [193]. While original sex-based differences in immunoglobulin and antibody levels persist, GAHT increases inflammatory markers and causes hormone-specific DNA methylation of immune genes. Even though the impact of these immune shifts on MS progression remains unclear, they emphasize the need for careful monitoring and detailed hormonal histories in transgender and gender-diverse people with MS.

### 3.2. Smoking and Air Pollution

In addition to being a risk factor for MS onset, smoking has also been shown to influence disease progression after MS diagnosis. Several studies, although not all, have reported an association between smoking and increased neurological disability [202,203,204] or conversion from CIS to definite MS or from relapsing-remitting MS (RRMS) to SPMS [205,206,207,208]. Each additional year of smoking after diagnosis was associated with a 4.7% faster progression to SPMS; therefore, smoking cessation is strongly recommended for all patients with MS [209].

Sex differences in the association between smoking and clinical outcomes have rarely been assessed.

Current smoking has been linked to a higher risk of developing neutralizing antibodies against interferon beta-1a or natalizumab, potentially impacting treatment response; however, no sex-related differences were observed for interferon beta, and such differences were not investigated for natalizumab [188,210].

Long-term exposure to air pollutants has been associated with increased relapse risk, and gadolinium-enhancing MRI lesion number, greater disability progression (EDSS), and higher hospitalization rates in MS patients [11,90]. In a Brazilian study, elevated SO_2_ levels were linked to a higher risk of hospitalization in female MS patients compared with males, while PM_10_ and NO were associated with increased risk only in women [189]. These findings support the hypothesis of sex-specific responses to air pollution, potentially mediated by hormonal interactions with stress and reproductive axes; however, the absence of adjustment for established MS risk factors limits the strength of the conclusions [189,211].

### 3.3. Obesity

In people with MS, obesity was linked to greater neurological disability, faster disease progression, reduced quality of life, higher relapse rate, accelerated cognitive decline, increased MRI activity and greater gray matter atrophy [212,213,214]. Smoking and obesity were shown to interact to adversely affect disease progression and cognitive performance, suggesting common pathological pathways leading to low-grade inflammation are shared between the two risk factors [215].

In animal models, obesity promoted Th1 inflammation and worsened EAE severity more in female mice than males: an increase in IFN-alfa and IFN-g/STAT1 signaling in mouse CD4^+^ T cells was proposed as a sex-specific mechanism to worsen central nervous system autoimmunity selectively in females [63]. A sex-specific association with disease severity was observed in obese MS patients: higher BMI was associated with higher cross-sectional EDSS in women, but with lower EDSS in men [190]. Female sex was associated with higher odds of rapid weight gain after MS diagnosis, even though no significant relationship was found between baseline disability and BMI change over time [191].

### 3.4. Vitamin D and Sun Exposure

Experimental and clinical studies have explored the relationship between vitamin D status, sun exposure, and MS disability progression. In EAE, administration of 1,25-dihydroxyvitamin D_3_ slowed or even reversed disability progression, suggesting a reversible blockade of disease activity [216]. Human observational studies have consistently linked higher serum 25(OH)D levels with slower disability progression, lower brain volume loss, and reduced relapse rates, while vitamin D deficiency has been independently associated with higher EDSS and MSSS [11]. Seasonal patterns in relapse occurrence, with peaks in winter, further support the influence of vitamin D status on disease activity [217].

While previous randomized controlled trials (SOLAR, CHOLINE, VIDAMS) and meta-analyses have not demonstrated significant benefits of high-dose vitamin D supplementation on relapse rate or disability progression in RRMS [218,219], a recent French trial demonstrated that oral cholecalciferol 100,000 IU every 2 weeks in monotherapy significantly reduced disease activity (both clinical and radiological) compared to placebo in CIS and early RRMS at 2-year follow-up [220]. Latest data affirm that vitamin D supplementation has produced modest but significant reductions in disability progression, relapses, and new T2-lesion formation [221], although it did not prevent conversion from CIS to MS [222,223].

Sex differences in the effect of vitamin D supplementation on myelination, microglial activation, apoptotic cell death and neuronal viability were assessed in an animal model of progressive MS [126]. In the absence of vitamin D supplementation, female mice exhibited stronger antioxidant defenses, reduced microglial activation and increased myelin preservation, with a delayed disease peak compared to males [126]. Vitamin D supplementation protected against neurodegeneration and oxidative stress in both sexes, with male mice exhibiting an even stronger protective effect than females, likely due to their lower baseline antioxidant defense capacity [126]. Vitamin D interacts with sex hormones to exert protective and therapeutic effects in MS, with estrogen–calcitriol synergy aiding recovery, while vitamin D and calcium jointly modulate immunity toward anti-inflammatory responses [224].

### 3.5. Microbiota and Diet

Gut microbiota composition is different in MS patients compared to healthy controls: bacterial products interact with the host immune system, driving either pro- or anti-inflammatory responses [225]. Gut microbiota composition has been linked to disability progression in MS: short-chain fatty-acid-(SCFA) producing bacteria (e.g., *Eubacterium hallii*, *Blautia*, *Roseburia*) were associated with slower progression and better clinical outcomes, whereas pro-inflammatory taxa such as *Alistipes*, *Streptococcus*, and *Clostridium* species correlated with greater disability and lesion burden [226,227]. Higher diet quality (rich in fruits, vegetables and grains, poor in processed meat and added sugar) and adherence to Mediterranean-style eating were associated with both objective and self-reported lower disability and better quality of life in MS [228,229,230,231,232]. For example, higher total consumption of lean and oily fish at diagnosis was associated with a reduced risk of confirmed disability worsening and reaching disability milestones compared with low consumption [233]. Lactobacillus and Bifidobacterium supplementation in cuprizone-induced demyelination rats mitigated oxidative stress, promoted remyelination, and enhanced vitamin D_3_ and B_12_ levels [234].

Gut microbial composition is influenced by genetics, diet, and sex hormones, which interact with one another [50]. In MS, the Mediterranean diet impacts the gut microbiota, increasing beneficial SCFA, thus promoting anti-inflammatory responses [225]. Gut microbial composition is different between sexes: the reciprocal interaction between gut microbiota and sex hormones has been demonstrated in several studies on healthy controls and mice [235]. Sex differences appear in the gut microbiota after puberty and sex hormones determine the species composition, independently of fecal transplantation [235]. At the same time, microbial metabolites interact with host cells through receptors (e.g., estrogen receptors) differentially expressed by sex, and also modulate estrogen and testosterone metabolism [50]. Gut microbial composition is different between sexes also in MS, with implications for intestinal and systemic immune responses [50,225]. In EAE models, estrogen therapy prevents EAE-associated dysbiosis of the microbiota and promotes the enrichment of bacteria that are associated with immune regulation [127]. Additional modifiers could also impact microbiota in a sex-specific manner: increased BMI alters the Firmicutes/Bacteroidetes ratio differently by sex, and probiotic supplementation elicits distinct inflammatory responses in male and female mice [235]. Sex-specific differences in the impact of diet on clinical outcomes in MS patients have not yet been evaluated.

### 3.6. Education

Socioeconomic factors, particularly higher educational attainment, have been inversely associated with disability progression in MS [236,237]. Both sociocultural education and genetic determinants of intelligence, as suggested by Mendelian randomization, have been linked to disability outcomes [238]. Maternal education was also found to influence disease severity, likely by shaping access to treatment and lifestyle risks [239]. According to the cognitive reserve hypothesis, higher education exerts a neuroprotective effect that enhances resilience and mitigates disability accrual [240]. However, the association between lower education and faster progression appears to be mediated by treatment and lifestyle factors [13], suggesting that education influences disability progression indirectly, primarily through its impact on health behaviors and treatment patterns. Despite a similar overall access to healthcare in Sweden, patients with only presecondary education were less likely to initiate second-line therapies, had shorter cumulative treatment exposure, and showed a higher prevalence of lifestyle risk factors such as smoking and reduced physical activity.

The inverse association between education and time to reach disability milestones was significant for both sexes in differential analysis, but was stronger in women with RRMS, who showed a clearer gradient in the risk of disability progression according to their level of education, especially in the early stages [192]. Interestingly, in the PPMS phenotype, differences in clinical outcomes were observed between very low and very high education groups in both sexes, but no gradient was detected. Patients with high-active cognitive reserve, assessed through Stern Leisure Activities, had more frequent RRMS and less commonly progressive MS, suggesting that loss of reserve may contribute to disease progression [241]. However, the role of education in shaping different disability trajectories and its interaction with sex remains to be clarified.

## 4. Implications for MS Diagnosis, Monitoring, and Treatment

Sex-related differences in MS have significant implications for diagnosis, disease monitoring, and therapeutic strategies.

### 4.1. Diagnosis

Recognizing sex- and lifestyle-related patterns in MS susceptibility is essential for timely and accurate diagnosis. The markedly higher prevalence of MS in women, particularly among those with early menarche, obesity, smoking exposure, or low vitamin D levels, calls for increased diagnostic vigilance during adolescence and reproductive age. Differences in the disease course in male patients, more likely to present with progressive phenotypes, poorer recovery after initial attacks, and faster accumulation of disability [92,102,103,107,109,111], should be taken into account to avoid diagnostic delays or misclassification, particularly in patients with non-relapsing or insidious presentations. In women, reproductive history, including menarche timing and parity, can offer important clues to risk and onset. In men, given the association with greater neurodegeneration and cognitive decline, targeted neuroimaging and neuropsychological assessment may support timely recognition of disease. Furthermore, integrating lifestyle and comorbidity profiles into the diagnostic framework, especially cardiovascular risk, depression, smoking, and obesity, can improve early risk stratification and help clinicians to anticipate disease trajectory from the earliest phases.

### 4.2. Monitoring

Both women and men’s hormonal status plays a significant role in modulating MS disease activity and progression. In women, reproductive transitions such as pregnancy, postpartum, breastfeeding, and menopause represent critical windows of immunological change that can either suppress or exacerbate disease activity. In men, low testosterone levels have been linked to worse cognitive and physical outcomes, with emerging evidence supporting a neuroprotective role for testosterone. These sex-specific hormonal dynamics highlight the need for individualized monitoring strategies that incorporate hormonal profiling, reproductive status, and sex-based patterns of relapse and progression, in order to more accurately predict disease course and optimize long-term management.

### 4.3. Treatment

Sex hormones are increasingly recognized as potential therapeutic targets. As estrogens exhibit both neuroprotective and immunomodulatory effects [8,91,92,121,125,242,243,244], reducing inflammatory lesions and improving cognitive function [91,174,197,245,246], selective ligands for estrogen receptor α and β have been proposed as therapeutic strategies [91,247,248,249,250,251]. Progesterone has demonstrated anti-inflammatory effects [91,92,128,252,253] and has been shown to promote remyelination in animal models [124,254,255,256]. In men or animal models, testosterone supplementation has demonstrated neuroprotective effects [8,123,185,186,248,257,258], including improvements in cognitive performance, reductions in brain atrophy, and increases in gray matter volume [91,97,123,185,186]. The TOTEM RRMS trial (NCT03910738) is an ongoing phase 2, placebo-controlled study in testosterone-deficient men with RRMS, testing whether testosterone undecanoate (vs. placebo) added to natalizumab over 66 weeks prevents progression through neuroprotective and remyelinating effects, with advanced MRI and clinical outcomes [259]. Indeed, subclinical testosterone deficiency may be underdiagnosed and could contribute to worse cognitive and functional outcomes. Although routine endocrine screening is not currently recommended in clinical practice, hormonal profiling may offer prognostic value and guide future neuroprotective strategies in men with MS.

Pregnancy and breastfeeding require careful management of disease-modifying therapies (DMTs) [9,93,195]. Many DMTs are discontinued during pregnancy, but certain injectable agents such as interferon-beta and glatiramer acetate are considered relatively safe through conception, gestation, and breastfeeding, particularly in women with low-to-moderate disease activity [9,93,195,260,261,262,263]. In clinical practice, natalizumab is frequently continued until around the 30th week of pregnancy, showing very favorable maternal clinical and radiological outcomes together with a reassuring fetal safety profile [264,265]. However, transient hematological alterations such as anemia or thrombocytopenia have been reported in infants exposed to natalizumab during the third trimester of pregnancy; therefore, a multidisciplinary team, including experienced pediatricians and pediatric hematologists, should attend the delivery in these specific cases, and the newborn’s blood counts should be closely monitored [266].

Even though avoiding pregnancy is still suggested for at least 4–6 months from the last ocrelizumab infusion, real-world data have reported no increased risk of major congenital anomalies, spontaneous abortion, or stillbirth for in utero ocrelizumab exposure [267,268,269]. To investigate whether ocrelizumab crosses the placenta or is excreted in breast milk with potential effects on infant B-cell development, immune function, and overall growth, two dedicated clinical studies—MINORE and SOPRANINO—have been initiated [270]. These trials aim to generate detailed pharmacokinetic, pharmacodynamic, and safety data, thereby providing valuable insights into the risk–benefit profile of ocrelizumab use during pregnancy and lactation in women with MS.

Short-half-life oral agents like dimethyl fumarate may be continued until pregnancy is confirmed, but are generally discontinued during breastfeeding [263]. Notably, recent data have reported favorable maternal and fetal outcomes in patients exposed to dimethyl fumarate during early pregnancy, supporting their cautious use in selected cases with high disease burden [271].

Conversely, other DMTs such as fingolimod, ozanimod, and ponesimod, as well as teriflunomide, remain contraindicated due to their teratogenic potential, and should be discontinued well in advance of conception, typically requiring a washout period of at least two months [263,272,273,274]. Newer agents such as cladribine and ponesimod lack robust reproductive safety data and are not recommended during pregnancy or breastfeeding [275]. However, cladribine, due to its pulsed dosing schedule and immune reconstitution profile, offers the advantage of allowing for pregnancy planning after a defined treatment-free interval, making it a potentially valuable option in patients planning pregnancy who require high-efficacy therapy [276,277].

Overall, DMT management during pregnancy should balance maternal disease control with fetal and infant safety; decisions must be individualized based on MS activity, drug safety profile, and breastfeeding plans, with multidisciplinary specialist involvement.

Finally, comorbidity management is critical, especially for conditions such as depression, cardiovascular disease, and hypertension, which can influence MS prognosis and exhibit sex-specific prevalence [11,278,279,280,281,282,283,284].

## 5. Conclusions

In summary, environmental and infectious factors such as smoking, obesity, vitamin D deficiency, ultraviolet radiation, and Epstein–Barr virus interact with sex-related biological pathways to shape multiple sclerosis risk. Women appear more susceptible to the pathogenic effects of smoking and obesity, but tend to benefit more from the protective influence of vitamin D and UV exposure, while EBV responses are further modulated by hormonal factors and host–virus genetic interactions. Disease progression also displays clear sex differences, with women experiencing greater inflammatory activity and men more often developing progressive neurodegenerative forms, influenced by reproductive phases, hormonal status, lifestyle factors, and social determinants. Understanding how hormonal, genetic, and environmental factors interact in a sex-specific manner is essential for developing individualized, effective, and safer therapeutic strategies. We hope that this overview of the existing evidence from animal and human studies on the impact of sex-related differences in lifestyle risk factors will encourage further research into sex differences in MS pathophysiology, disease course and treatment response, with the potential to drive innovation in personalized medicine for both women and men.

## Figures and Tables

**Table 1:** Sex-related differences in lifestyle factors affecting MS susceptibility (studies on animal models or human cells).

Risk Factor	Model/Cell Number	Sex-Specific Effect	Reference
Vitamin D	B10.PL-H2uH2-T18a/SnJ, C57BL/6 mice	VitD_3_ protects only intact females; no effect in males/ovariectomized unless E_2_ given. Synergy: VitD_3_ ↑ E_2_ synthesis, E_2_ ↑ VitD receptor in CNS → explains female bias in MS.	Nashold 2009 [17]
	Consomic mice (B6 vs. PWD)	High VitD protective only in female B6, not in males or resistant PWD. Effect via sex- and genotype-specific suppression of pro-inflammatory CD4^+^ T cell programs. Suggests gene–sex interactions.	Krementsov 2018 [18]
EBV	464 EBV^+^ B cells (GEUVADIS dataset)	Estradiol treatment alters MS-risk gene expression, proliferation, EBV DNA load and EBNA2 expression in a sex-dependent way.	Keane 2021 [19]
	69 EBV-infected B cells from 13 RRMS	Host genetic susceptibility and EBV genetic variability converge on shared, sex-dependent mechanisms relevant to MS pathogenesis.	Mechelli 2025 [20]

↑ increase, → as a consequence.

**Table 2:** Sex-related differences in lifestyle factors affecting MS susceptibility (studies on human subjects).

Risk Factor	Study Population	Sex-Specific Effect	Reference(s)
Smoking	394 MS, 394 HC	↑ MS risk in women (OR 6.48, 95% CI 1.46–28.78), not in men (OR 0.72).	Alonso 2011 [21]
	Birth cohorts, multi-country	MS sex ratio correlates with smoking sex ratio (r = 0.16, *p* = 0.002).	Palacios 2011 [22]
	109 MS, 218 HC	↑ Cotinine is linked to MS risk mainly in women (OR 3.9, 95% CI 1.3–12).	Sundström 2008 [23]
	356 MS	Men smoke more cigarettes than women (*p* = 0.024) and have longer duration before onset (9.2 vs. 4.5 yrs, *p* = 0.002).	Ashtari 2025 [24]
Passive smoking	129 MS, 1038 HC	Parental smoking ↑ MS risk (RR 2.12, 95% CI 1.43–3.15); no sex effect.	Mikaeloff 2007 [25]
Obesity (pediatric/adolescence)	75 MS/CIS [26]; 774 MS [27]; 453 MS [28]; 254 MS 420 HC [29];	Higher BMI ↑ MS risk only in girls (*p* = 0.005) [26], in both sexes, but more in girls [28]; ↑ BMI z-score (7–13 years) ↑ risk in girls (HR 1.18–1.20); weaker in boys [27]; ↑ postpubertal BMI ↑ MS risk in girls OR 1.60 and boys OR 1.43. Earlier puberty → earlier MS onset (girls: *p* = 0.031; boys: *p* < 0.001) [29].	Langer-Gould 2013 [26]; Munger 2013 [27]; Huppke 2019 [28]; Chitnis 2016 [29]
Obesity (women)	593 MS	Obesity at 18 years doubles MS risk (RR 2.25, 95% CI 1.50–3.37, *p* < 0.001).	Munger 2009 [30]
Obesity (men)	628 MS, 6187 HC	No association of BMI or parental occupation with MS risk in men.	Gunnarsson 2015 [31]
Vitamin D	103 MS, 110 HC	Each +10 nmol/L 25(OH)D ↓ MS odds in women by 19% (OR 0.81, 95% CI 0.69–0.95).	Kragt 2009 [32]
UVR	2667 MS	Prevalence inversely correlated with UVR: stronger in females (r = −0.76, *p* < 0.001) vs. males (r = −0.46, *p* = 0.032).	Orton 2011 [33]
EBV	259 IM/MS	IM→MS delay shorter in males/high EBNA1, longer in females/low EBNA1 (shortening beyond puberty).	Endriz 2017 [34]
Puberty	5493 MS, 1759 HC	No association between puberty timing and MS risk in males. In females, earlier puberty increases MS risk (RR = 0.9 per year later, *p* = 0.00017). No effect on age at onset.	Ramagopalan 2009 [35]
	150 MS	Earlier menarche associates with earlier MS onset: age at first symptoms increases by 1.16 years for each year delay in menarche (R^2^ = 0.69, *p* = 0.04).	Sloka 2006 [36]
Oral contraceptive	315 MS/238371 [37]; 1131 MS/181058 [38], 891MS/3564 HC [39]	No association with MS risk [37,38,39]; in nulliparous women, longer or earlier OC use ↑ MS risk 2–3x [38]	Hernán 2000 [37]; Nova 2024 [38]; Zhang 2024 [39]
	106 MS	MS incidence is 40% lower in OC users versus nonusers (OR 0.6, 95% CI 0.4–1.0)	Alonso 2005 [40]
	400 MS/CIS, 3904 HC	OC use ↑ MS/CIS risk (OR = 1.52, 95% CI = 1.21–1.91; *p* < 0.001)	Hellwig 2016 [41]

↑ increase, → as a consequence or to, ↓ decrease.

**Table 3:** Sex-related differences in lifestyle factors affecting MS progression (studies on animal models).

Risk Factor	Model	Sex-Specific Effect	Reference
Hormonal exposure	Wild-type and transgenic mice [120], or B-cell-deficient mice [121], ARNesCre mice [122], castrated male C57BL/6 mice [123], Female C57Bl/6 mice [124] Female SJL/J, male C57BL10/J mice [125]	E2 and progesterone enhance astrocyte numbers and promote remyelination [120,124]. E2 protective effects are lost in B-cell-deficient mice (no reduction in disease severity) [121]. Testosterone is required for spontaneous remyelination in male mice, depending on CXCR4 signaling [122] and preserves excitatory synaptic transmission in the hippocampus [123]. Estriol, but not progesterone treatment, reduces the severity of EAE [125].	Bardy- Lagarde 2025 [120], Bodhankar 2011 [121], Asbelaoui 2024 [122], Ziehn 2012 [123], El-Etr 2016 [124], Kim 1999 [125]
Obesity	C57BL/6J, SJL/J, and db/db mice	Obesity promoted stronger Th1 inflammation and worsened EAE severity in females, linked to IFN-α/γ–STAT1 signaling in CD4^+^ T cells.	Cordeiro 2024 [63]
Vitamin D	Dark Agouti rats (46 females, 50 males)	Females had higher antioxidant defenses, myelin preservation, and delayed disease peak; however, vitamin D supplementation conferred greater benefit in males due to lower baseline antioxidant capacity.	Haindl 2024 [126]
Gut microbiota	Female C57BL/6 mice	Pregnancy-level estrogen reshaped gut microbiota, prevented EAE-related changes, and enriched immune-regulatory bacteria, suggesting hormone–microbiota cross-talk in neuroprotection.	Benedek 2017 [127]

**Table 4:** Sex-related differences in lifestyle factors affecting MS progression (studies on human subjects).

Risk Factor	Study Population	Sex-Specific Effect	Reference
Pregnancy	254 MS [128], 355 pregnancies [129], 338 MS [130], 61 MS [131], 28 MS [132], 2 MS [133], 2105 MS [134], 677 [135], 2466 MS [136], 501 MS [137], 973 MS [138], 137 MS, 396 HC [139]	Relapse rates drop up to 70% in late pregnancy [128,129,130,131], with frequent post-partum clinical/radiological rebound [128,129,130,131,132,133]. Pregnancy may slow disability progression [134,135,136,137,138], with parous women reaching milestones later, with a pregnancy-dose effect [139].	Confavreux 1998 [128], Hellwig 2012 [129], Korn-Lubetzki 1984 [130], Jalkanen 2010 [131], Paavilainen 2007 [132], Van Walderveen 1994 [133], Ramagopalan 2012 [134], Karp 2014 [135], Jokubaitis 2016 [136], Zuluaga 2019 [137], D’hooghe 2012 [138], Zeydan 2020 [139]
Breastfeeding	254 MS [128], 201 MS [140], 375 MS [141], 140 MS [142], 173 MS [143], 867 MS [144], 32 MS, 29 HC [145], 350 pregnancies [146], 108 MS [147], 61 MS [148], 93 MS [149], MS [150], 97 MS [151], 43 MS, 21 HC [152], 210 MS [153]	Breastfeeding, especially exclusive, reduces postpartum relapse risk [140,141,142,143,145,153]; non-exclusive breastfeeding compared to exclusive breastfeeding associates with higher relapse risk [140] Breastfeeding does not associates with relapses [128,146,147,148,149,150,151,152], early post-partum treatment with natalizumab/fingolimod reduces relapses compared with exclusive breastfeeding [144].	Confavreux 1998 [128], Hellwig 2015 [140], Langer-Gould 2020 [141], Gulick 2002 [142], Haas 2007 [143], Haben 2024 [144], Langer-Gould 2009 [145], Portaccio 2014 [146], Achiron 2004 [147], Airas 2010 [148], Benoit 2016 [149], De Las Heras 2007 [150], Jesus-Ribeiro 2017 [151], Runia 2015 [152], Lorefice 2022 [153]
Menopause	412 MS [154] 724 MS [155], 147 MS [156], 75 MS [157], 559 MS [158], 148 MS [159], 184 MS [160], 37 MS [161]	Menopause associates with reduced ARR [156,161], increased disability progression [155,159], greater atrophy [154,156], greater MSFC worsening [154,160] and rapid increase in sNfL [160]. Disability progression remains stable after menopause [157,158,161].	Graves 2018 [154], Bove 2016 [155], Lorefice 2023 [156], Otero-Romero 2022 [157], Simonsen 2025 [158], Baroncini 2019 [159], Silverman 2025 [160], Ladeira 2018 [161]
Hormonal replacement therapy	14 MS, 13 HC [162], 248 MS [163], 16 MS, 15 HC [164], 3325 MS [165]	HRT improves quality of life [163]. HRT does not associate with disability accrual (trend toward higher risk with longer use) [165]; it does not change sGFAP or sNfL levels [164] nor MRI activity [162].	Juutinen 2022 [162], Bove 2016 [163], Juutinen 2024 [164], Kopp 2022 [165]
Assisted reproductive technologies	3 MS/1 CIS [166], 6 MS [167], 23 MS [168], 36 MS [169], 115 MS [170], 65 MS [171], 225 MS [172]	GnRH agonists may increase relapse/MRI activity [166,167,168,169]. No increased relapse risk with either GnRH agonists or antagonists [170,171,172].	Laplaud 2006 [166], Hellwig 2008, 2009 [167,168], Michel 2012 [169], Mainguy 2025, 2022 [170,172], Graham 2023 [171]
Oral contraceptives	973 MS [138], 202 MS [173], 12 MS [174], 495 MS [175], 132 MS [176], 164 MS [177], 1210 MS [178], 174 MS [179], 150 MS [180], 46 MS [181], 62 MS [182]	OC associate with higher risk of reaching disability milestones [138]. No efficacy in preventing postpartum relapses [173]. OC do not negatively affect long-term prognosis [174,175,176]. OC delay PIRA [178] and associate with lower disability [176,179,182], MRI activity [180] or relapses [177]. Continuous OCs show a trend towards less inflammatory activity on MRI relative to cyclic OCs [181].	D’hooghe 2012 [138], Vukusic 2021 [173], Sicotte 2002 [174], Otero-Romero 2022 [175], Sena 2012 [176], Voskuhl 2016 [177], Giordano 2025 [178], Gava 2014 [179], Pozzilli 2015 [180], Chen 2020 [181], Ferret-Sena 2024 [182]
Testosterone	96 MS men [183] 10 MS men [184,185,186]	Testosterone levels in men negatively associate with clinical and cognitive outcomes [183]. Testosterone therapy slows gray matter [186] and brain atrophy rate and improve cognition [185] via immune modulation and increased neurotrophic factors [184].	Bove 2014 [183], Sicotte 2007 [185], Gold 2008 [184], Kurth 2014 [186]
Gender-affirming hormone therapy	17 TGD-MS	In transgender individuals with MS, GAHT increases clinical and radiological activity	Neyal 2025 [187]
Smoking	695 MS	Smoking ↑ risk of NAbs to IFNβ-1a (OR 1.9, *p* = 0.002); no sex differences.	Hedström 2014 [188]
Air pollutants	2490 MS hospitalizations	Exposure to an increase of 1 µg/m^3^ in SO_2_ concentration associates with a hospitalization risk increase +10% in females, +7.5% in males. PM_10_ and NO associate with ↑ risk only in females.	Diniz 2023 [189]
Obesity	1037 MS	Higher BMI was associated with higher cross-sectional EDSS in women, but with lower EDSS in men (*p* = 0.003, N = 758).	Bove 2016 [190]
	924 MS	Education protective from weight gain (OR 0.88); rapid weight gain (≥2 kg/m^2^/year) more frequent in females than males (OR 1.84); disability not associated with annualized BMI increases.	Conway 2025 [191]
Education	11,586 MS	Higher education ↓ risk of EDSS 4 in RRMS at 5 years in both sexes; stronger effect in women (HR 0.39–0.74) vs. men (HR 0.29–0.63).	Lefort 2025 [192]

↑ increase, ↓ decrease

## Data Availability

No new data were created or analyzed in this study. Data sharing is not applicable to this article.

## Abbreviations

The following abbreviations are used in this manuscript:

ART	Assisted reproductive technologies
BMI	body mass index
CIS	clinically isolated syndrome
DMTs	disease-modifying therapies
EAE	experimental autoimmune encephalomyelitis
EBNA	Epstein–Barr nuclear antigen
EBV	Epstein–Barr Virus
EDSS	Expanded Disability Status Scale
GAHT	gender-affirming hormone therapy
GnRH	gonadotropin-releasing hormone
HRT	hormonal replacement therapy
IM	infectious mononucleosis
IVF	in vitro fertilization
MS	Multiple Sclerosis
MSSS	Multiple sclerosis severity score
OC	oral contraceptives
PIRA	progression independent of relapse activity
RRMS	relapsing-remitting MS
SCFA	short-chain fatty-acid
SPMS	secondary progressive MS
Tregs	regulatory T cells
UVR	ultraviolet radiation
25(OH)D	25-hydroxyvitamin D
